# Regional Behavior of Airspaces During Positive Pressure Reduction Assessed by Synchrotron Radiation Computed Tomography

**DOI:** 10.3389/fphys.2019.00719

**Published:** 2019-06-07

**Authors:** Gaetano Scaramuzzo, Ludovic Broche, Mariangela Pellegrini, Liisa Porra, Savino Derosa, Angela Principia Tannoia, Andrea Marzullo, Joao Batista Borges, Sam Bayat, Alberto Bravin, Anders Larsson, Gaetano Perchiazzi

**Affiliations:** ^1^Department of Morphology, Surgery and Experimental Medicine, University of Ferrara, Ferrara, Italy; ^2^Hedenstierna Laboratory, Department of Surgical Sciences, Uppsala University, Uppsala, Sweden; ^3^The European Synchrotron Radiation Facility, Grenoble, France; ^4^INSERM UA7, Synchrotron Radiation for Biomedicine (STROBE) Laboratory, Grenoble Alpes University, Amiens, France; ^5^Department of Anesthesia and Intensive Care, Uppsala University Hospital, Uppsala, Sweden; ^6^Department of Physics, Faculty of Mathematics and Natural Sciences, University of Helsinki, Helsinki, Finland; ^7^Helsinki University Central Hospital, Helsinki, Finland; ^8^Department of Emergency and Organ Transplant, University of Bari Aldo Moro, Bari, Italy; ^9^Centre for Human and Applied Physiological Sciences, Faculty of Sciences and Medicine, King’s College London, London, United Kingdom

**Keywords:** recruitment, VILI, alveoli, kinetics, SRCT

## Abstract

**Introduction:**

The mechanisms of lung inflation and deflation are only partially known. Ventilatory strategies to support lung function rely upon the idea that lung alveoli are isotropic balloons that progressively inflate or deflate and that lung pressure/volume curves derive only by the interplay of critical opening pressures, critical closing pressures, lung history, and position of alveoli inside the lung. This notion has been recently challenged by subpleural microscopy, magnetic resonance, and computed tomography (CT). Phase-contrast synchrotron radiation CT (PC-SRCT) can yield *in vivo* images at resolutions higher than conventional CT.

**Objectives:**

We aimed to assess the numerosity (ASden) and the extension of the surface of airspaces (ASext) in healthy conditions at different volumes, during stepwise lung deflation, in concentric regions of the lung.

**Methods:**

The study was conducted in seven anesthetized New Zealand rabbits. They underwent PC-SRCT scans (resolution of 47.7 μm) of the lung at five decreasing positive end expiratory pressure (PEEP) levels of 12, 9, 6, 3, and 0 cmH_2_O during end-expiratory holds. Three concentric regions of interest (ROIs) of the lung were studied: subpleural, mantellar, and core. The images were enhanced by phase contrast algorithms. ASden and ASext were computed by using the Image Processing Toolbox for MatLab. Statistical tests were used to assess any significant difference determined by PEEP or ROI on ASden and ASext.

**Results:**

When reducing PEEP, in each ROI the ASden significantly decreased. Conversely, ASext variation was not significant except for the core ROI. In the latter, the angular coefficient of the regression line was significantly low.

**Conclusion:**

The main mechanism behind the decrease in lung volume at PEEP reduction is derecruitment. In our study involving lung regions laying on isogravitational planes and thus equally influenced by gravitational forces, airspace numerosity and extension of surface depend on the local mechanical properties of the lung.

## Introduction

Lung inflation and deflation have been described by pressure–volume (PV) curves measured at the airway opening. Its sigmoidal shape, delimited by inflection points (Venegas et al., 1998), would derive from a sequence of prevalent alveolar dynamics, with recruitment at low volumes followed by isotropic inflation and then hyperinflation at high volumes (Amato et al., 1995; Roupie et al., 1995). Many clinical studies have been proposed to titrate mechanical ventilation settings using information provided by PV curves directly (Amato et al., 1998) or assuming its course in proportion to body weight (Acute Respiratory Distress Syndrome Network et al., 2000).

However, an “inherent inhomogeneity” of regional lung mechanics has been demonstrated (Crotti et al., 2001; Pelosi et al., 2001), suggesting the existence of multiple asynchronous events of alveolar recruitment and inflation through the whole inspiratory portion of the PV curve (Hickling, 1998). This has been also demonstrated in healthy lungs (Perchiazzi et al., 2011). Moreover, experimental data suggest that together with alveoli that expand during the inspiration (opening, distending, or hyper-distending) there are other alveoli that contract (Schiller et al., 2003; Kaczka et al., 2011; Perchiazzi et al., 2014). This phenomenon was mostly detected with small volume increments (Perchiazzi et al., 2014).

The relationships between gas inhalation and exhalation at airway opening as well as the modalities of airspace inflation and deflation are not fully known. Two plausible alveolar inflation mechanics have been suggested (and may co-exist): (A) isotropic balloon behavior, i.e., increasing of gas volume in proportion to gas delivery (“analog model”) and (B) open or closed behavior, i.e., having room for a defined amount of gas, which does not change in direct proportion to the overall volume change. Pursuant to B the lung inflates/deflates due to multiple simultaneous recruitment/derecruitment events (“digital model”).

At the current state of knowledge the relative roles of alveolar recruitment and alveolar change in shape and size are not fully understood and are largely disputed. Considering that one of the potential mechanisms of ventilator-induced lung injury (VILI) (Tremblay and Slutsky, 2006) is the co-existence of airspaces with different time constant in close proximity, the microscopic inflation and deflation mechanisms need to be better investigated.

Such current uncertainty about lung microscopic behavior derives partially from the methods of investigation. Microscopic observations of *ex vivo* lungs provide high resolution images but they are distant from physiological conditions (Perlman and Bhattacharya, 2007). *In vivo* subpleural microscopy allows to study lungs at high resolution, but only in its outer surface and with modified local external forces on lung surface (Carney et al., 1999; Salito et al., 2014). Computed tomography (CT) (Gattinoni et al., 2001; Hajari et al., 2012) and He-hyperpolarized nuclear magnetic resonance (NMR) allow imaging the entire lung but with limited spatial resolution to identify individual airspaces.

To our knowledge there is no data available of lung airspaces that fulfill all the following conditions: *in vivo*, high spatial resolution, and access to internal structures of the lung. Phase-Contrast Synchrotron Radiation CT (PC-SRCT) allows enhancing contrast in weakly X-ray-absorbing tissues as is the case in the lung. This imaging technique uses highly coherent monochromatic radiation with a long sample to detector distance to measure interferences between refracted and direct radiation. Synchrotron X-ray sources are particularly suited by providing such characteristics, then allowing for rapid acquisition times for spatial resolutions permitting the assessment of the lung morphological details.

The main aim of this study was to assess the mechanisms through which healthy lungs microscopically deflate, by quantifying the number and extent of airspaces at decreasing volumes during a stepwise lung deflation maneuver and in different concentric regions of interest (ROIs) of the lung.

## Materials and Methods

Care and handling of the animals involved in this scientific research followed the Directive 2010/63/EU of the European Parliament (European Union, 2010). The effective procedures were reviewed and approved by the Internal Evaluation Committee for Animal Welfare in Research of the European Synchrotron Radiation Facility (ESRF, Grenoble, France) and were executed in accordance to the principles expressed by the ARRIVE (*Kilkenny et al., 2010*) and the PREPARE guidelines (*Smith et al., 2018*).

### Experimental Setup

Seven male New Zealand rabbits (3.8 ± 0.4 kg) underwent general anesthesia after the positioning of a 22 G catheter into the marginal ear vein under local anesthesia; anesthesia was induced by i.v. injection of sodium thiopental (25 mg/kg) and maintained by IV midazolam (0.2 mg/kg/h) and atracurium (1.0 mg/kg/h) after ensuring an adequate depth of the anesthesia plan. After tissue infiltration of local anesthesia, the animals were tracheotomized using a Portex tracheal tube (n.3, Smiths Medical, Kent, United Kingdom). A central venous catheter and an arterial line were placed via surgical dissection, respectively, in the left jugular vein and into the ipsilateral carotid artery for fluid/drugs administration and for arterial pressure monitoring. Mechanical ventilation was delivered by a standard Servo-I ventilator (Getinge-Maquet, Solna, Sweden). The ventilator was connected by wire to a standard Maquet interface placed in the control room. Baseline ventilation was delivered in pressure-controlled ventilation mode, with a positive end expiratory pressure (PEEP) of 3 cmH_2_O and a plateau pressure titrated to obtain a tidal volume (*V*_T_) of 6 ml/kg; I:E ratio was 1:2, F_I_O_2_ = 0.6. Respiratory rate was initially set at 40 bpm and then regulated to obtain a PaCO_2_ between 35 and 45 mmHg.

Respiratory flow was measured by a heated pneumotachograph (Hans Rudolph, Kansas City, MO, United States); volumes were computed by integrating flow signals. Pressure in the airways, together with flow and arterial pressure, were continuously sampled at 4 kHz onto a dedicated data acquisition system (Powerlab, ADInstruments, Oxfordshire, United Kingdom) and recorded on a laptop computer.

### Synchrotron Radiation Computed Tomography

The experiments were conducted at the Biomedical Beamline ID 17 of the ESRF (Grenoble, France). The animal was immobilized in upright position and met a stationary X-ray beam while rotating on its vertical axis. The broad-spectrum synchrotron radiation was filtered by a double-crystal Si monochromator (Suortti et al., 2000) to obtain a narrow energy band around 65 keV.

After the sample, X-rays are converted into visible light by a 20-μm thick gadolinium oxysulfide scintillator, the photons are then recorded by a Fast Readout Low Noise Charge Coupled Device (CCD) camera (FReLoN, ESRF, Grenoble, France) with a 2048 × 2048 pixel chip (Coan et al., 2006).

The optical system, in its whole, produced an effective voxel size of 47.7 μm^3^. To obtain a complete CT scan, 1000 angular projections were necessary during expiratory pauses. Reconstruction of images was executed by a filtered back-projection algorithm using the HST program, residing on the ESRF GPU clusters (Mirone et al., 2014). Eventual ring artifacts were corrected using a wavelet interpolation using in house software. Before reconstruction, images were filtered using the Paganin algorithm (Paganin et al., 2002) which allows to highlight signal originating from tissues of different composition, when images are acquired using the propagation phase contrast imaging setup, like in this case on the base of the geometry and the approximate elemental composition of the tissue.

### Study Protocol

After a stabilization period of 30 min following instrumentation, we performed a volume history normalization maneuver, consisting in the application of a continuous positive airway pressure (CPAP) of 20 cmH_2_O for 20 s. Then, PEEP was set to 12 cmH_2_O and SRCT images were acquired during expiratory pauses. Between the exposures to SRCT, the animals were ventilated (as during the stabilization phase) with a *V*_T_ of 6 ml/kg. The same imaging sequence was performed at PEEP levels of 12, 9, 6, 3, and 0 cmH_2_O. Plateau pressure was not allowed to exceed 38 cmH_2_O; whenever it happened, the *V*_T_ was reduced before the corresponding step of SRCT exposure.

### Image Recording and Analysis

The animals were studied in vertical position; during each recording session, 40 adjacent SRCT images were acquired, each one containing an iso-gravitational slice of the lung. In order to comply with the limitations of the vertical size of the beam when necessary the imaging sequence could be repeated by vertically displacing the sample.

**FIGURE 1:**
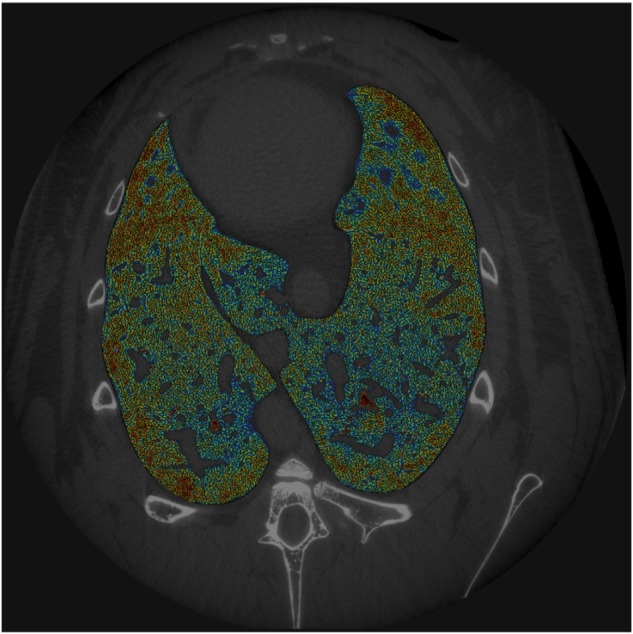
Example of SRCT image. For each animal, 40 sequential SRCT images were obtained from the central part of the lungs. Images were processed using the phase-contrast technique (see text for further details). Airspaces color is the function of air content.

On each studied slice, after a manual segmentation of the entire lung parenchyma boundary, we applied a sequence of *Top-Hat* transforms (Dougherty and Lotufo, 2003) differing for the dimension of the structuring element, which transforms images into binary matrices and delimitates objects inside the SRCT slices (Figure 1). We defined as “airspaces” the areas of the SRCT images that contained gas, according to the physical density of their content. These were anatomically separated from adjacent airspaces by septal-like structures showing a tissue-like density.

**FIGURE 2:**
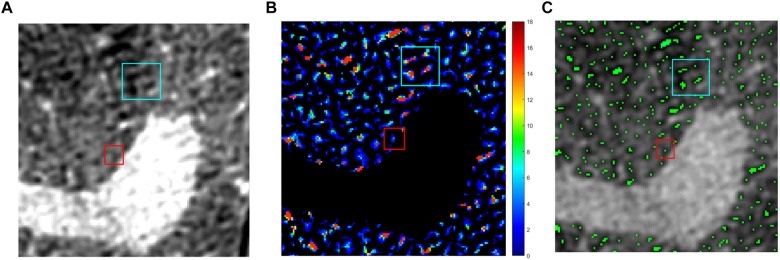
Image processing. Example of image processing. Multiple top-hat processes combined with the automatic identification of regional peaks allowed to count the NAs. **(A)** Original image, **(B)** multiple top-hat processes, and **(C)** regional peaks.

**FIGURE 3:**
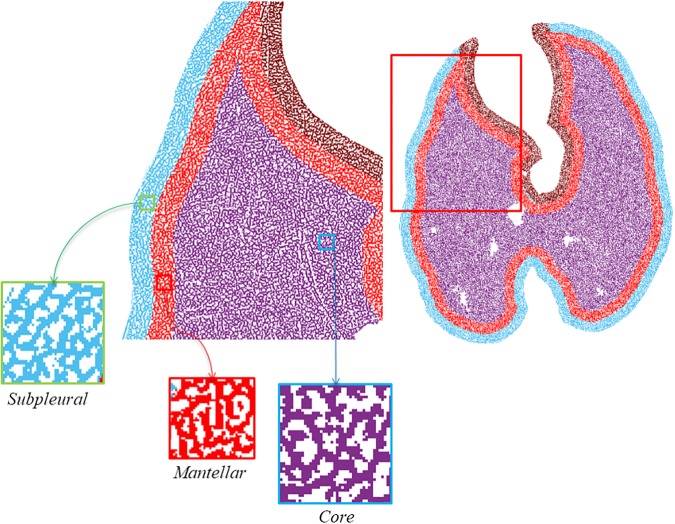
Definition of regions of interest (ROIs). The brown area corresponding to the parenchyma around the heart has been excluded from the analysis to eliminate the motion artifacts connected to the heart activity.

The obtained binary image was studied as whole (labeled as ALL) and as divided into three concentric ROIs labeled as subpleural, mantellar, and core ROI, starting at, respectively, 0, 2, and 4 mm from the pleural surface (Figure 2, 3). All the procedures of image analysis were performed by using MatLab Image Processing Toolbox by using purposely developed scripts written by two of the authors (GS and GP).

The superimposition of the different binary images deriving from multiple *Top-Hat* transforms allowed to identify local peaks corresponding to the airspaces. The number of airspaces (NAs) was calculated counting the local negative intensity peaks applying a custom-made MATLAB script. The NAs were calculated for each of the three ROIs (subpleural, mantellar, and core regions of the lung) and in the 40 consecutive slice levels sampled in each animal at each PEEP level. The density of airspaces in each ROI was expressed as units per mm^3^ (ASden). The volume subtended by each ROIs was calculated knowing that each SRCT voxel is a cube having a side of 47.7 μm (see also Supplementary Material).

We calculated the total area covered by airspaces as the total surface (the number of the pixels multiplied for their surface they covered) inside the perimeter of tissue-density boundaries, as derived from the above described image segmentation procedure. The sum of all the areas coming from the 40 consecutive layer surfaces yielded the value of the surface covered by airspaces in the analyzed lung region.

Dividing the surface covered by airspaces for the NAs we obtained the average airspaces surface extension (ASext) according to the following equation (Eq. 1):

(1) $$\mathrm{Airspaces average surface extension}=\frac{\sum_{k=1}^{40}\mathrm{Surface covered by airspaces}\;(k)}{\sum_{k=1}^{40}\mathrm{Number of airspaces}\;(k)}.$$

### Data Analysis

We studied the effect of PEEP on the NAs (ASden) and on the *average dimension* of airspaces (ASext); the analysis was conducted on a global (the entire analyzed section, i.e., from the subpleural space to the mediastinum) and on a regional basis (subpleural, mantellar, and core regions). To test if the applied PEEP influenced ASext and ASden we performed a linear regression analysis. We compared the significant regressions equations by applying the *F*-test to evaluate whether the effect of PEEP was different in the different ROIs.

The Wilcoxon signed-rank test was used to test differences between continuous variables. The set α-value was 0.05 in all the statistical tests and was purposely corrected according to Bonferroni (1936) if any of the test involved multiple comparisons.

## Results

The animals survived the experimental protocol. In all animals, the driving and plateau pressures varied according to the respective compliance of the respiratory system subtended by the existing lung volume at each step of the study protocol (Table 1).

**Table 1:** Respiratory parameters.

Variable	PEEP 12	PEEP 9	PEEP 6	PEEP 3	ZEEP
*V*_T_ (ml)	28.8 ± 10.7	29.9 ± 10	30.1 ± 9.9	31.5 ± 12.8	31.8 ± 11.8
PEEP (cmH_2_O)	12.3 ± 0.5	9.5 ± 0.6	6.9 ± 0.8	4 ± 0.9	1.2 ± 0.4
Ppeak_rs_ (cmH_2_O)	32.7 ± 6.2	23.4 ± 2.4	18.3 ± 1.8	16.5 ± 3.7	14.2 ± 2.7
Pplat_rs_ (cmH_2_O)	31.4 ± 6.5	18.7 ± 1.9	16.6 ± 0.3	13.03 ± 1	13.3 ± 2.3
Driving pressure (cmH_2_O)	20.4 ± 6	13.9 ± 2	11.4 ± 1.6	12.5 ± 3	13 ± 2.6
C_rs_ (ml/cmH_2_O)	1.5 ± 0.5	2.1 ± 0.5	2.6 ± 0.5	2.5 ± 0.5	2.4 ± 0.6

*Ventilatory data recorded during the protocol at the different levels of PEEP. Data are expressed as mean ± SD.*

### Airspaces Number and Dimension in the Whole Lung Slice

The ASden of the entire parenchyma progressively decreased while reducing PEEP (Table 2), passing from 233.5 ± 15.6 at PEEP 12 to 216.7 ± 15.2 at PEEP 0. Their ASext decreased also in proportion to the applied PEEP level: from 17.3 ± 1.1 at PEEP 12 to 16 ± 2 at PEEP 0. The linear regressions between applied PEEP versus ASext and ASden were statistically significant (*p* < 0.05).

**Table 2:** Results.

								Linear Regression			

		ROI	PEEP 12	PEEP 9	PEEP 6	PEEP 3	PEEP 0	*m*	*k*	*R*^2^	*p*
Entire slice	*ASext* (*voxels*)	ALL	17.3 ± 1.1	17.4 ± 1	17.3 ± 1	17.1 ± 1	16 ± 2.1	0.11	16.3	0.09	<0.01
	*ASden* (*n/mm^3^*)	ALL	233.5 ± 15.6	230.6 ± 14	229.1 ± 14.4	224.7 ± 14.3	216.7 ± 15.2	1.47	217.01	0.14	<0.01
Regional analysis	*ASext* (*voxels*)	SUB	16.6 ± 0.8	17 ± 0.9	16.8 ± 1	16.8 ± 1	16 ± 1.9	0.05	16.26	0.04	0.3
		MAN	16.9 ± 0.7	16.9 ± 0.7	16.9 ± 0.7	16.8 ± 0.8	15.8 ± 1.9	0.09	16.07	0.1	0.06
		COR	18.3 ± 1	18.3 ± 0.9	18.1 ± 0.9	17.8 ± 1	16.2 ± 2.7	0.18	16.56	0.19	<0.01
	*ASden* (*n/mm^3^*)	SUB	245.8 ± 8.4	239 ± 9.4	238.2 ± 10.5	234.7 ± 8.8	223.3 ± 12.7	1.86	223.6	0.39	<0.01
		MAN	239.4 ± 7.3	238.9 ± 5.1	236.9 ± 5	231.8 ± 5.7	224.9 ± 10.9	1.33	225.39	0.38	<0.01
		COR	215.4 ± 9.5	214 ± 7.5	212.1 ± 7.3	207.7 ± 7.5	201.9 ± 10.5	1.21	202.04	0.27	<0.01

*Airspaces extension (ASext) and density (ASden) at different levels of PEEP [reported as (cmH_2_O); data expressed for the whole analyzed slice and for the three regions of interests (SUB, subpleural; MAN, mantellar; COR, core]. Values expressed as mean ± SD.*

### Airspaces Number and Dimension in the Three ROIs

**FIGURE 4:**
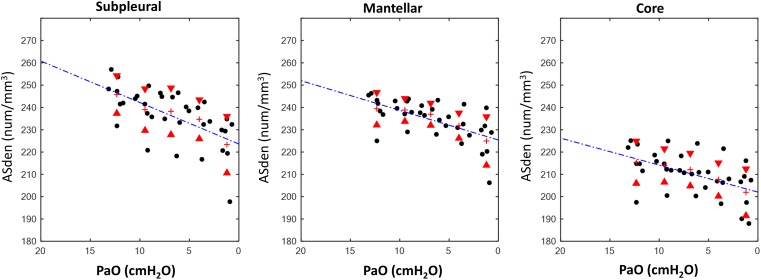
Results, airspaces density (ASden). Relationship between measured positive end expiratory pressure (PaO) and airspaces density (ASden, num/mm^3^) in the different analyzed ROIs.

**FIGURE 5:**
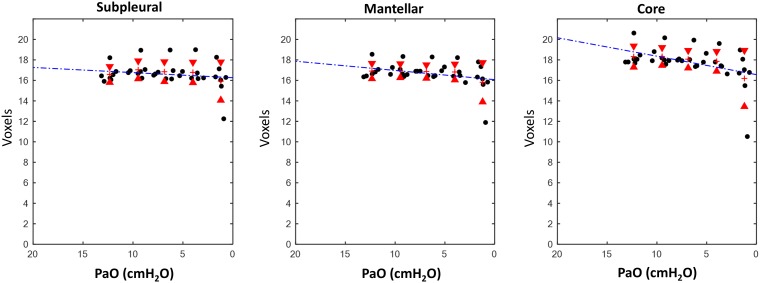
Results, airspaces dimension (ASext). Relationship between measured PEEP (PaO) and airspaces dimension, expressed in voxels (1 voxel = 47.7 μm) in the different analyzed ROIs.

The results for the regional analysis are reported in Table 2. The linear regression analysis showed a significant decrease of ASden in all ROIs when decreasing PEEP. An opposite pattern was found for ASext: this parameter, with the exception of the core region, did not statistically change. In the latest ROI (core area of the lung) the angular coefficient *m* of the correlation line was markedly low (Figure 4, 5).

### Effects of ROI Location on Airspaces Density and Dimension

The effect of ROI location is summarized in Supplementary Table S1 (Supplementary Material). The core ROI had a significantly lower ASden in all applied PEEP compared to the subpleural and mantellar ROIs. ASext was significantly smaller in the core ROI than in the subpleural and in the mantellar ROIs with the exception of PEEP 0 and 3 cmH_2_O. No difference was found between mantellar and subpleural ROIs in terms of both ASext and ASden.

## Discussion

The present experimental study, based on synchrotron radiation CT, aimed at analyzing airspaces microscopic behavior in healthy lungs. Both peripheral and core regions of the lung were explored *in vivo* during controlled mechanical ventilation during a decremental PEEP trial.

The main findings of our study in healthy lungs are the following: (1) the reduction of lung volume at end-expiration during a decremental PEEP trial was mainly related to a decrease of the airspaces number and not of their dimensions; (2) the distance from the pleural space influenced the airspaces behavior, i.e., in the core region both dimension and number changed significantly, with a predominance of the latter.

### Number and Dimensional Variation

Our findings suggest that if the airspace is open it maintains a stable surface extension while the regional lung volume is changing. This behavior was evident when examining the single ROIs. In the core both ASden and ASext decreased with PEEP reduction but, although statistically significant, the ratio of this change was very low (angular coefficient = 0.18). The other two ROIs (subpleural and mantellar) did not show a statistically significant decrease of the surface extension. Considering the whole slice (labeled as ALL in Table 2), we were not able to detect a predominant behavior between recruitment and inflation as in the single ROIs. We hypothesize that, despite of being exposed to the same superimposed pressure, the different ROIs are subjected to different local traction forces as from the fixed vessels, bronchi, and related to the subpleural space outward. Therefore, pooling airspaces together cannot demonstrate specific deflation behaviors.

The decision of defining concentric ROIs was also taken to compare the results of the well-known subpleural microscopy techniques with our data. Our findings within the subpleural region are in line with the ones by Carney et al. (1999) who found, using subpleural microscopy, an increase in the number of alveoli as the main responsible for lung volume increases from a degassed state to 80% of total lung capacity. With their experimental setup, alveolar volume changed significantly only from 10 to 20% of TLC. Hajari et al. (2012) studied human lung inflation with ^3^He-MRI and concluded that “in healthy human subjects the lung inflates primarily by alveolar recruitment and, to a lesser extent, by anisotropic expansion of alveolar ducts.” This conclusion is coherent with our results likely because they studied the lungs as a whole, without differentiating subpleural and core regions, as we did in our pooled analysis (Table 2, under the label “ALL”). However, Hajari et al. (2012) evaluated the mechanisms of lung inflation whereas our study evaluated the deflation behavior of the lung. Escolar et al. (2002) performed a morphometric study using light microscopy on rat lungs and concluded that “changes in lung volume are related to the increase/decrease in the number of alveoli that are open/closed and not to the modification in the size of the alveoli.”

It has been postulated that the main mechanism by which the recruitment is progressive is the stochastic distribution of the critical opening and closing pressures of the airspaces, making the airspaces open or closed (Crotti et al., 2001) in a sequential way (Bates, 2007). It is disputed if the distribution of the critical opening and closing pressures within the lung parenchyma is Gaussian (Crotti et al., 2001) or linear (Albert et al., 2009). Similarly, it is debated whether reaching the critical opening/closing pressures is the only mechanism involved in the processes of PEEP inflation/deflation of airspaces. It is plausible that an important role is played by the presence or not of fluid bridges inside the small airways, which could influence the processes of inflation/deflation (Gaver et al., 1990; Otis et al., 1993). In addition, the propagation of the processes of inflation and deflation can be also facilitated by sequential avalanche mechanisms once the critical opening pressures within the airways with bigger caliber are reached (Suki et al., 1994).

### Regional Differences

At each PEEP level and for both airspace numerosity and average surface, subpleural and mantellar airspaces exhibited non-different behaviors. The core ROI, instead, exhibited a behavior that was statistically different from the mantellar and subpleural ROIs, although it was reported by a seminal study that subpleural and internal alveoli are subjected to the same transmural pressures (Mead et al., 1970). The core ROIs, when compared to the two more superficial ROIs, were probably more affected by local forces created by the network of vessels, bronchi, and connective tissue (Rausch et al., 2011). It is worth noting that the three ROIs followed the same qualitative pattern, i.e., inflation by recruitment mechanisms.

These observations altogether suggest that it is questionable to make general inferences on number and average surface of airspaces by observing the lung from the outer surface.

### Physiological and Clinical Implications

The search for how to titrate/individualize a PEEP level for critically ill patients on a timely and physiology-based fashion is still awaiting better answers. One of the major challenges is the lack of a precise and detailed understanding of the effects of a volume change in the lung parenchyma. The aim of our study was to add relevant and new understanding on how the pulmonary parenchyma responds, microscopically, to decreases in volume at decremental PEEP, both in superficial and core regions. Our methods enabled us to evaluate the lung *in vivo* both within superficial and core parenchymal regions, without opening the chest wall and modifying the forces applied to the alveolar surface.

We purposely studied the deflation behavior instead of the inflation one. This choice derives from the will of reproducing the clinical maneuver used to set PEEP. In fact, the PV curve of the lung has a hysteretic behavior (Bachofen et al., 1970). There is evidence that only a decremental PEEP titration (Borges et al., 2006a,b) can determine accurately the lung specific closing pressures and consequently the optimum PEEP level capable of maintaining lung functional residual capacity (FRC). In doing so, it is possible to oppose repeated derecruitment-associated lung injury, even in normal lungs (Borges et al., 2017). By maintaining lung FRC, PEEP can also contribute in reducing cyclic recruitment–derecruitment (Muscedere et al., 1994), preserving surfactant function (Verbrugge et al., 1998), thereby minimizing the related shear stress (Otto et al., 2008).

The term “recruitment” has been classically used to justify an unexpected increase of respiratory system compliance in relation to volume history (Mead and Collier, 1959) or applied PEEP levels (Falke et al., 1972). Successively, this wording has been used to describe the appearance of ventilated areas during compartmental image analysis (Gattinoni et al., 1987). In more recent times, this phenomenon has been considered as a potential cause of VILI (Pinhu et al., 2003) when it occurs cyclically during the tidal ventilation (*atelectrauma*). In the present paper we use the term recruitment to indicate the phenomenon of airspace opening as a mechanism of lung inflation by PEEP during artificial ventilation. Further studies are necessary for confirming this evidence also during spontaneous breathing or lung diseases. Moreover, the *atelectrauma* involves also terminal airways cyclic opening/closing (Broche et al., 2017), that till now has not been identified during mechanical ventilation in healthy conditions. Indeed, tidal opening and closing of distal bronchioles (“bronchiolotrauma,” Borges, 2018) might also play a role as a triggering factor in a potentially hazardous chain of events during patient self-inflicted lung injury (Brochard et al., 2017). The weight of the *atelectrauma* in the pathophysiology of ALI deserves more studies (Gattinoni et al., 2018) even in healthy lungs, since there is an increasing call for protective ventilation also during anesthesia (Borges et al., 2017).

### Technical Aspects and Limitations

We have used throughout the manuscript the term “airspace” to indicate areas of the parenchyma which contain gas and are surrounded by structures having tissue-like density. It is known that the alveoli of the rabbit have an average diameter of 110 μm (Tenney and Remmers, 1963; D’Angelo, 1972), more than the double of the SRCT pixel size used in the present experiments which is 47.7 μm. A reported peculiarity of the rabbit lung is that distal bronchioles can have very few alveoli (McLaughlin et al., 1966) or none (Cruise and Brewer, 1994) but instead terminate in vestibules (alveolar ducts) which contain the most of alveoli. Moreover, these alveolar ducts can be very large in diameter, and reach their maximum dimensions at the lobar and costophrenic angles where they can measure up to 0.5–0.75 mm. Thus, the lung mechanical properties could be different from humans. Furthermore, we used an inspired concentration of oxygen that might increase the tendency for derecruitment. Since the resolution of our images was 47.7 μm, we were not able to efficiently delineate alveolar boundaries because the average dimension of inter-alveolar walls is well below this limit.

We applied a linear function as a model of regression between the applied PEEP and the NAs and between the applied PEEP and the *average surface* of airspaces. Although different nonlinear models could have been used, we observed that in reference studies (Bachofen and Hilderbrandt, 1971; D’Angelo, 1972; Gil et al., 1979) on similar issues, in the range of pressure that we used in the present study (between 0 and 12 cmH_2_O) the behavior of the spatial displacement of the lung can be assumed to be linear.

We present a study based on an animal model and conducted in a specific laboratory setting: whether these finding can be extended to other species or to humans must be confirmed by further studies. In fact, mechanical ventilation is not a natural condition: in this case ventilation is based on the generation of a positive pressure at the airways opening and not, as during spontaneous breathing by the generation of a negative pressure by the inspiratory muscles. This different arrangement of the forces that concur to the generation of transpulmonary pressure does not allow to infer any conclusion on the airspace dynamics during natural spontaneous breathing on the base of the present experiments.

On the other side, a possible study of airspaces during spontaneous breathing at high resolution is technically demanding. In fact there are several experimental issues with this kind of studies regarding mainly the maintenance of a suitable depth of anesthesia and an adequate technical setup able to trace and image the naturally irregular respiratory rate.

Our results showed a decrease of the alveolar number with PEEP reduction while the animals were paralyzed with muscle relaxants. Consequently on the base of our experimental setup we cannot differentiate the roles of the lack of muscular tone from the sole absence of inspiratory efforts. So we could not exclude that the presence of muscle tone (in absence of inspiratory efforts) could have contributed to maintain the alveoli open and to which extent.

In our model, for technical reasons, we were obliged to place the animals in a vertical position. Usually ICU patients lay in supine position and isogravitational planes are parallel to the coronal planes. When studying the lung with a clinical CT machine, the obtained sections are transversal and so exposed differently to gravitational forces. The weight of the lung structures in the nondependent portions exerts a force on the dependent part, particularly when the lung is affected by pathologies that modify its density. This superimposed pressure alters the alveolar opening pressures and affects the process of lung inflation (Gattinoni et al., 1991; Pelosi et al., 1994; Pellegrini et al., 2016). In our experimental model, the tissue imaged in the single CT slices lays on the same gravitational plane, allowing us to assume that the superimposed pressure was the same in all the parts of it. Moreover, for the same technical reasons mentioned above, in our experimental set-up we could not image the lung in supine position. For all these reasons (images shot in vertical position and impossibility to obtain images from the supine position to compare with), no conclusion could be drawn about the specific effects of vertical positioning on alveolar kinetics.

In the present experiment we have not studied the process of edema formation. In preceding preparatory pilot experiments we observed its formation when the heart has a failing performance and mainly in the most dependent areas of the lungs. Edema formation can play an important role both in the process of gas exchange and in modifying the above mentioned superimposed pressure acting on lung parenchyma.

As all the image analysis computation procedures, this sequence of calculations also can be affected by a systematic error in precision, especially when the thickness of the delimitating borders of alveoli is below the limit of resolution. This is the reason of using the term “airspace” and the avoidance of using a more specific anatomic terminology. However, the structure of rabbit lung, terminating into vestibules on which the most of alveoli directly open, let us consider these units as a whole, both anatomically and functionally. In fact, although ducts and alveoli are distinct anatomical structures and in principle might have different mechanisms for opening and closing, actually in literature there is no evidence that the “mother-duct” has a different opening or closing pressure than its “son-alveoli.” On the contrary, several studies on lung injury have demonstrated a simultaneous involvement of the entire unit: membranous bronchioles and alveolar ducts (Muscedere et al., 1994) or distal airway unit (Tsuchida et al., 2006). These evidences favor the idea that the response to a change of pressure is simultaneous in the entire distal structure, supporting the concept that our definition of *airspaces* is representative of the behavior of both alveoli and ducts.

For sake of correctness, it must be stated that if a bias on the computation of the number and/or the surface area of the airspaces is potentially seen, it affects all the slices and all the measurements. In other words, although the absolute values of the parameters might be affected, their trend is not, maintaining the validity of the results of our study.

## Conclusion

Our data suggest that the macroscopic decrease in end expiratory lung volume during a decremental PEEP trial is related to a reduction of the NAs more than to their reduction in volume, especially in the subpleural lung. At low PEEP levels, a reduction in volume was also present within the core lung.

## Contribution to the Field Statement

The experiments reported in this manuscript were devoted to understand the mechanism of lung deflation when a patient that is mechanically ventilated in the intensive care unit undergoes a reduction of the set PEEP. The clinical relevance of the proposed study lies in the fact that the downward stepwise reduction of PEEP is commonly used for deciding the best PEEP at which the patient has to be ventilated for several hours. In consideration that mechanical ventilation *per se* may induce the so called “VILI” syndrome, because of heterogeneous transmural pressure gradients (developed by the diseased lungs), knowledge of lung airspace dynamics may allow to avoid particularly injurious patterns of breathing.

## Data Availability

The datasets generated for this study are available on request to the corresponding author.

## Ethics Statement

This study was carried out in accordance with the recommendations of Directive 2010/63/EU of the European Parliament (European Union, 2010). The effective procedures were reviewed and approved by Internal Evaluation Committee for Animal Welfare in Research of the European Synchrotron Radiation Facility (Grenoble, France).

## Author Contributions

GP, AL, JB, and SB conceived the study and the protocol. LB, MP, LP, SD, SB, AL, JB, and GP carried out the experiment and collected the data. GS, AM, GP, and AT analyzed the data. GP carried out the statistical analysis. GS and GP wrote the manuscript and all the authors participated to its revision.

## Conflict of Interest Statement

The authors declare that the research was conducted in the absence of any commercial or financial relationships that could be construed as a potential conflict of interest.
