# Supplementary material for: Regional Behavior of Airspaces During Positive Pressure Reduction Assessed by Synchrotron Radiation Computed Tomography
Source: Front Physiol. 2019 Jun 7;10:719. doi: 10.3389/fphys.2019.00719 (PMC6567926; doi:10.3389/fphys.2019.00719)
Supplement: Supplementary file 1 [file Table_1.DOCX]

**SUPPLEMENTAL MATERIAL**

**About the method**

We used a propagation-based phase-contrast computed tomography^1^ that can enhance the contrast of images deriving from tissues like lungs whose components (mainly water and air) weakly absorb x-rays. In fact, this technique profits of the long distance (7 meters) between sample and detector: the highly coherent monochromatic radiation coming from the synchrotron source, while crossing the sample, yields two simultaneous images, one produced by linear transmission of unabsorbed X rays and the other by the processes of X-ray deviation due to matter scattering and refraction^1-3.^

The Top-Hat function requires some parameters to enhance the contrast for objects boundaries discrimination. The most sensitive parameter in this segmentation process is the thickness of the boundary to be found. Since in our analysis boundaries are defined as alveolar/airspaces septum and since they do not have a constant thickness and a sharp definition at this resolution.

On each image, we applied sequentially multiple Top-Hat functions differing only for the dimension of the structuring element in order to comply with objects separated by boundaries of different thickness (dimensions used: 1-17 pixels). We defined as airspaces the areas of the SRCT images that contained gas, according to the physical density of their content. These were separated from adjacent airspaces by septal-like structures showing a tissue-like density. From this enhancement technique we obtained binary images with boundaries containing the structural element; using structural elements differing for their dimensions, we could detect different type of boundaries.

The reason for this image post-processing is that in our images, in fact, the spatial resolution did not allow to clearly identify alveoli; we therefore indirectly counted alveoli by counting the regional negative peak related to local air content. Further studies at higher resolution are needed to confirm our findings.

**Bibliography**

1. Wilkins, S. W., Gureyev, T. E., Gao, D., Pogany, a. & Stevenson, a. W. Phase-contrast imaging using polychromatic hard X-rays. *Nature* **384,** 335–338 (1996).
2. Coan P, Peterzol A, Fiedler S, Ponchut C, Labiche JC, Bravin A. Evaluation of imaging performance of a taper optics CCD; FReLoN' camera designed for medical imaging. J Synchrotron Radiat. 2006 May;13(Pt 3):260-70. Epub 2006 Apr 13.
3. Bravin A, Coan P, Suortti P. X-ray phase-contrast imaging: from pre-clinical applications towards clinics. Phys Med Biol. 2013 Jan 7;58(1):R1-35. doi: 10.1088/0031-9155/58/1/R1. Epub 2012 Dec 10.

Supplemental Table S1: Multiples comparison between ROIs using Wilcoxon signed-rank test; α=0.05, p significant if <0.05/n according to Bonferroni correction for n simultaneous comparisons


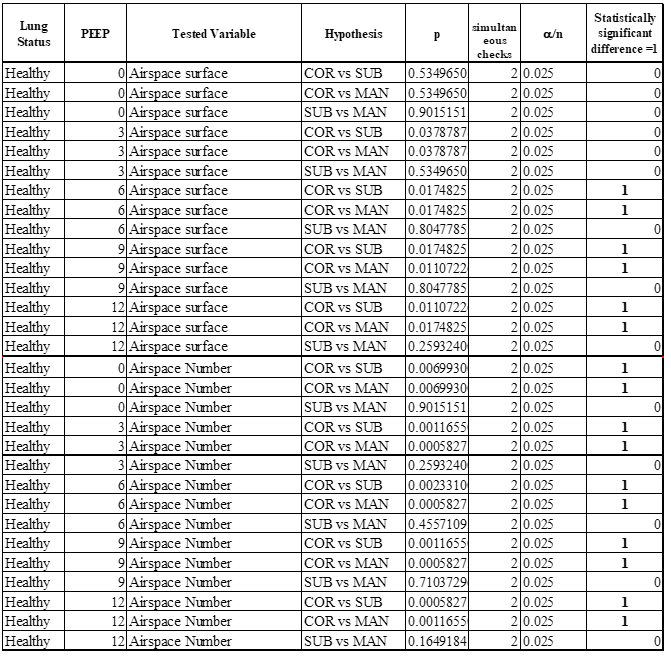


.
